# Deoxynivalenol Determination Using Innovative Lateral Flow Device Technology

**DOI:** 10.3390/toxins17030123

**Published:** 2025-03-05

**Authors:** Delphine Halberstadt, Paola Giorni, Erica Barato, Terenzio Bertuzzi

**Affiliations:** 1 R-Biopharm AG, An der Neuen Bergstraße 17, 64297 Darmstadt, Germany; d.halberstadt@r-biopharm.de; 2 Department of Sustainable Crop Production (DIPROVES), Università Cattolica del Sacro Cuore, 29122 Piacenza, Italy; paola.giorni@unicatt.it; 3 Department of Animal Science, Food and Nutrition (DIANA), Faculty of Agricultural, Food and Environmental Science, Università Cattolica del Sacro Cuore, 29122 Piacenza, Italy; erica.barato@unicatt.it

**Keywords:** deoxynivalenol, lateral flow device technology, LC-MS/MS analysis

## Abstract

Deoxynivalenol (DON), generally the most widespread mycotoxin in wheat, is regulated by the EU regulation in cereals and cereal-derived products. Its presence can be detected by chromatographic or rapid methods; the latter technique is generally used in control analysis, fulfilling the needs of the stakeholders of the wheat grain chain. Lateral flow strips are often used for the rapid detection of different mycotoxins in several agricultural products; regarding DON determination, different lateral flow immunochromatography strips are currently available, also providing quantitative results. The purpose of this work was to evaluate the accuracy of an innovative lateral flow device coupled to a bench top device, following a digital approach. The proposed method was compared to an LC-MS/MS method, analyzing 50 naturally contaminated wheat samples. The results obtained using the two methods were very similar and, applying a paired *t*-test, the mean difference between measurements resulted not significantly different (α = 0.003). The correlation between the results showed a slope of the line close to 1 (m = 0.9904) and a regression coefficient (r) of 0.9968.

## 1. Introduction

Trichothecenes (TCTs) may occur in cereal grains because of *Fusarium* head blight (FHB), which is associated with a complex of the *Fusarium* species [1,2]. TCTs are classified into two groups: type A and type B TCTs. Type B TCTs, produced mainly by *F. graminearum* and *F. culmorum*, are more common and include deoxynivalenol (DON), which is generally the most widespread mycotoxin in wheat produced in Italy [3,4,5]. Soft and durum wheat are very important crops in Italy; soft wheat is cultivated mainly in northern Italy (about 75%), while durum wheat is prevalent in central and southern Italy. Previous studies reported that wheat from northern Italy is often contaminated with DON; on the contrary, wheat produced in central and southern Italy shows generally low contamination [6,7]. TCTs are implicated in several human health problems because of their stability during food processing and can cause serious health risks to animals consuming contaminated feed [8,9,10]. Recently, the European Commission [11] lowered the maximum permissible level for DON in unprocessed soft and durum wheat destined for human consumption to 1000 and 1500 µg/kg, respectively. For animal nutrition, pigs are very sensitive to DON occurrence in feed and a maximum value of 0.9 mg/kg was indicated in the EU recommendation [12].

Due to the EU regulation, all the stakeholders of the wheat grain chain must analyze the cereal lots for DON quantification. The presence of DON in cereals and cereal-based products can be detected by chromatographic methods like high-performance liquid chromatography (HPLC or UHPLC) coupled with ultraviolet (UV), diode array (DAD), or mass spectrometry (MS) detectors, and gas-chromatography (GC) coupled with electronic capture (ECD) or MS detectors [13,14,15,16,17,18]. These chromatographic techniques ensure high sensitivity, accuracy, and reproducibility, but are expensive and require personnel with specialized training. Moreover, the control analyses must be fast when grain is delivered from farmers to storehouses; therefore, rapid methods are generally used since they fulfill the needs of the two parts involved, acquiring and delivering actors. The first rapid methods were ELISA tests, based on an antigen–antibody reaction. For DON determination, competitive enzyme immunoassay is generally used, where free DON and DON enzyme conjugates compete for the DON antibody binding sites. Finally, the measurement made at 450 nm is inversely proportional to DON concentration, achieving very low detection limits. Recently, several rapid methods based on different techniques, such as colorimetric biosensors, hyperspectral imaging, visible near-infrared spectroscopy, magnetic aptasensors, and lateral flow immunoassay were developed obtaining interesting results [19,20,21,22,23,24,25,26,27]. Referring to rapid analytical methods that provide qualitative or semi-quantitative results, lateral flow immunochromatography strip (LFD) is a promising technique with several advantages, such as low cost, simplicity, sensitivity, speediness, and high specificity. To date, lateral flow strips are often used for the rapid detection of different mycotoxins in several agricultural products. The fast screening of undesired compounds through the cereal chain allows for the reduction of the occurrence of contaminated grains, preventing contaminated lots from entering the storage/processing step. This technique allows for the reduction of the waiting time and high cost of chromatographic analyses, which are only required to confirm the samples positive at screening using rapid methods [28].

Regarding DON determination in cereals, different LFDs are currently available, also providing quantitative results. The quantification is carried out using a specific anti-DON antibody which detects DON in the sample extract. During the incubation of the test strip, a band pattern (test line/control line) forms; the concentration of DON is determined by the evaluation of these lines using a dedicated detector.

The purpose of this work is to evaluate an innovative LFD technique based on the combination of a lateral flow device with a software application using a bench top device for evaluating DON contamination in wheat lots following a digital approach. This approach allows for the simplification of the quantification step and to store and share the results. To evaluate the accuracy of the proposed method, a comparison with an LC-MS/MS analysis was carried out in several naturally contaminated wheat samples.

## 2. Results

### 2.1. Validation Parameters of a RIDA^®^QUICK DON Method Combined with the RIDA^®^SMART APP

After analyzing a large number of uncontaminated samples as reported in the Section 4, the limit of detection (LOD) was calculated as the mean value + 3 times standard deviation, while the limit of quantification (LOQ) was calculated as the mean value + 9 times standard deviation (Table 1). Comparing the three lots, LOD and LOQ values resulted different; this variability can be due to the fact that LFDs are generally developed to be accurate at concentrations close to the legal limits and are not able to quantify low values, such as ELISA kits or chromatographic methods. Our results confirmed that this technique cannot be accurate at levels lower than the legal limit. Then, we chose to consider LOD and LOQ as the highest values of three lots. For the following measurements, the LOQ was fixed to 0.250 mg/kg.

The accuracy of the method was evaluated by analyzing twelve certified wheat samples (Trilogy Analytical Laboratories, Washington, MO 63090 USA) using two different lots of LFD; three replicates for each sample were analyzed. The recovery values resulted between 82% and 114% in comparison to contamination given by Trilogy, fulfilling the range of recovery for analytes at trace levels (Table 2).

### 2.2. Comparison Between RIDA^®^QUICK DON RQS ECO in Combination with the RIDA^®^SMART BOX and the RIDA^®^SMART APP and the LC-MS/MS Method

The naturally contaminated samples analyzed using LC-MS/MS showed a range of contamination from <0.050 (LOQ) to 15.48 mg/kg (Table 3). Six samples were contaminated at levels below 0.050 mg/kg. Using the RIDA^®^SMART BOX method, the LOQ was fixed to 0.250 mg/kg and a total of ten samples resulted contaminated below this value. Moreover, three certified naturally contaminated wheat samples (Trilogy) were analyzed. Their reported concentration level was 0.5, 1.9, and 4.3 mg/kg; the results found using the LC-MS/MS and RIDA^®^SMART BOX methods were 0.46 and 0.38 mg/kg, 1.78 and 1.89 mg/kg, 4.44 and 4.10 mg/kg, respectively. Table 3 shows the contamination levels obtained using the two methods.

**Figure 1:**
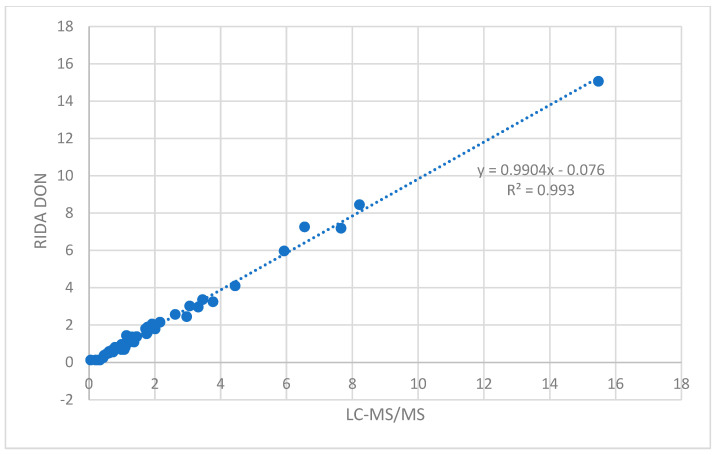
Correlation between DON contamination levels (mg/kg) obtained using LC-MS/MS and RIDA^®^QUICK DON RQS ECO with the RIDA^®^SMART APP methods.

The results were very similar; the correlation between the results showed a slope of the line close to 1 (m = 0.9904) and a regression coefficient (r) of 0.9968 (Figure 1); for uncontaminated samples, a value of 0.025 and 0.125 mg/kg was assigned using the LC-MS/MS and RIDA^®^SMART BOX methods, respectively. Applying a paired *t*-test, the mean difference between measurements resulted not significantly different (α = 0.003).

## 3. Discussion

Since testing and quantifying cereal lots for DON is necessary along the entire food supply chain, the requirement is often to generate fast and accurate results, mainly for the contaminants’ quantification. Chromatographic methods ensure high sensitivity, accuracy, and reproducibility, but are expensive, time intensive, and need a purification step. Moreover, qualified personnel are required. The lateral flow immunochromatography strips are fast and simple, less expensive, and no qualified operators are required. The combination of a lateral flow device with the RIDA^®^SMART APP is a new technology of quantification with the possibility to export and send the results easily anywhere following a digital approach. This technology gives several advantages, since the APP can be installed on a validated Smartphone or used in combination with the RIDA^®^SMART BOX. Using the RIDA^®^SMART BOX, the strip only needs to be placed in the BOX and the quantitative evaluation can be started in the APP. The results can be directly reported out of the APP via email to any cloud or Wi-Fi printer. Moreover, the analysis of certified samples and the comparison of naturally contaminated samples with the LC-MS/MS method demonstrated very satisfactory accuracy and precision not only at levels close to the legal limit, but also in a large range of contamination. 

## 4. Materials and Methods

### 4.1. Sampling

A total of fifty wheat samples (both soft and durum) collected in 2024 in northern Italy were analyzed using an LC-MS/MS method and an innovative LFD technique called RIDA^®^QUICK DON RQS ECO (R-Biopharm AG, 64297 Darmstadt, Germany), with final quantification using the RIDA^®^SMART APP in combination with the RIDA^®^SMART BOX (R-Biopharm). A total of fifteen different wheat varieties was considered. The samples were milled using a cyclone miller hammer to pass a 1 mm sieve, homogenized, and kept at −20 °C until the analysis. For the parameter validation of the LFD method, DON was determined in several certified wheat samples provided by Trilogy Analytical Laboratories (Washington, MO 63090 USA).

### 4.2. Sample Preparation and Test Procedure for DON Determination Using the RIDA^®^QUICK DON RQS ECO Lateral Flow Device with the RIDA^®^SMART APP and the RIDA^®^SMART BOX

DON was extracted by mixing 5 g of a ground and homogenized sample with 25 mL distilled or deionized water. This was shaken for 30 s and then centrifuged for 1 min 2000× *g*, filtered, or waited to be settled down. A volume of 100 µL of the extract was diluted with 500 µL of mobile solvent and thoroughly mixed by inverting the tube. A volume of 100 µL of this dilution was pipetted onto the application area of the test strip and incubated for 3 min. Then, the result was evaluated using the RIDA^®^SMART APP Software (Version 1) in combination with the RIDA^®^SMART BOX (Version 1).

### 4.3. Validation of the RIDA^®^QUICK DON RQS ECO Lateral Flow Device with the RIDA^®^SMART APP

The limit of detection (LOD) and the limit of quantification (LOQ) were determined by testing 36 wheat samples (both soft and durum). This resulted in contamination at levels below 0.20 mg/kg using the chromatographic method and coming from the countries of Italy and Germany. All samples were extracted three times and tested with three different test kit lots of RIDA^®^QUICK DON RQS ECO according to the instruction for use of the test kit (test procedure see below); a total of 324 extracts were analyzed.

The accuracy and precision were carried out by testing Trilogy^®^ wheat quality control material. The identified DON values of the Trilogy^®^ materials given on the certificates were set as target values (100%). Twelve wheat materials were extracted three times and tested with two lots of RIDA^®^QUICK DON RQS ECO according to the instruction for use of the test kit. The results were evaluated with the RIDA^®^SMART APP software installed on a validated smartphone. The mean results and recovery rates as well as the coefficient of variation (CV) for the RIDA^®^QUICK DON RQS ECO measurements are shown in Table 2.

### 4.4. Sample Preparation for DON Determination Using LC-MS/MS Analysis

From an aliquot of 25 g of milled wheat, DON was extracted using 100 mL acetonitrile: water 80 + 20 *v*/*v*. The extraction was carried out for 60 min using a rotary shaker., After filtration through a filter paper, the extract was diluted with the mixture methanol:water 10 + 90 *v*/*v* (200 µL extract + 400 µL mixture) before LC-MS/MS determination (Vanquish pump and autosampler coupled with Fortis mass spectrometer, Thermofisher). Chromatographic separation was carried out using a Betasil RP-18 column (5 µm particle size, 150 × 2.1 mm, Thermo-Fisher, Waltham, MA, USA) with a mobile-phase gradient methanol-Ammonium Acetate 10 mM (pH 6.8) from 10:90 (isocratic 2 min) to 65:35 in 4 min, then isocratic for 3 min, gradient to 10:90 in 1 min, and isocratic for 6 min (conditioning step). The ionization was carried out with an H-ESI interface (Thermo-Fisher) in negative mode as follows: spray capillary voltage 3.1 kV, sheath and auxiliary gas 35 and 15 psi, respectively, vaporizer temperature 200 °C, and temperature of ion transfer tube 270 °C. The fragmentation ions were 247, 265, and 295 *m*/*z* (parent ion 355 *m*/*z*, adduct with acetate), collision gas (Argon) was 1.5 psi, and the collision energy was among 12 and 16 V. The limit of detection (LOD) and the limit of quantification (LOQ) were 20 µg/kg and 50 µg/kg, respectively.

### 4.5. Data Analysis

Results obtained using the two analysis methods were statistically compared applying a paired T-test to verify the mean difference between measurements [29,30]. The statistical package IBM SPSS Statistics (Version 27) (IBM Corp., Armonk, NY, USA) was used for this analysis.

## Figures and Tables

**Table 1:** LOD and LOQ of RIDA^®^QUICK DON RQS ECO with the RIDA^®^SMART APP.

	No of Total Tests	Mean Value [mg/kg]	SD	LOD [mg/kg]	LOQ [mg/kg]
Lot 1	n = 324	0.092	0.011	0.125	0.190
Lot 2		0.049	0.010	0.080	0.142
Lot 3		0.124	0.013	0.162	0.239

**Table 2:** Recovery of Trilogy material RIDA^®^QUICK DON RQS ECO with the RIDA^®^SMART APP.

Trilogy^®^control ^a^	D-W-1207 ^b^ (0.5 mg/kg)			D-W-159 (0.7 mg/kg)			D-W-177 (1.6 mg/kg)		
	mg/kg	Recovery	CV ^c^	mg/kg	Recovery	CV	mg/kg	Recovery	CV
Lot 1	0.57	114%	8.0%	0.67	96%	0.9%	1.64	102	3.0%
Lot 2	0.51	103%	8.8%	0.65	92%	5.4%	1.61	100	5.6%
Trilogy^®^control	D-W-193 (2.3 mg/kg)			D-W-192 (2.9 mg/kg)			D-W-169 (3.5 mg/kg)		
	mg/kg	Recovery	CV	mg/kg	Recovery	CV	mg/kg	Recovery	CV
Lot 1	2.27	99%	4.6%	2.74	95%	2.1%	3.50	100%	2.2%
Lot 2	2.31	100%	3.5%	2.46	85%	8.7%	3.24	92%	5.2%
Trilogy^®^control	D-W-176 (4.0 mg/kg)			D-W-196 (5.4 mg/kg)			D-W-179 (8.9 mg/kg)		
	mg/kg	Recovery	CV	mg/kg	Recovery	CV	mg/kg	Recovery	CV
Lot 1	3.77	94%	8.2%	5.40	100%	4.7%	9.15	103	6.1%
Lot 2	3.55	89%	5.1%	4.92	91%	7.2%	8.45	95	6.7%
Trilogy^®^control	D-W-197 (9.3 mg/kg)			D-W-189 (28.9 mg/kg)			D-W-1208 (36.3 mg/kg)		
	mg/kg	Recovery	CV	mg/kg	Recovery	CV	mg/kg	Recovery	CV
Lot 1	9.00	97	3.2%	24.83	86	1.4%	29.75	82	1.5%
Lot 2	7.80	84	6.7%	27.63	96	6.1%	35.42	98	13.5%

^a^ Trilogy^®^control: product group (control material) of the company Trilogy. ^b^ D-W-1207: labeling of the naturally contaminated control material. ^c^ CV: coefficient of variation.

**Table 3:** DON contamination levels (mg/kg) in 50 naturally contaminated samples collected in northern Italy determined using LC-MS/MS and RIDA^®^QUICK DON RQS ECO with the RIDA^®^SMART APP in combination with the RIDA^®^SMART BOX methods (Kit LFD).

Sample	LC-MS/MS	Kit (LFD)	Sample	LC-MS/MS	Kit LFD ^a^
1	8.22	8.45	26	<0.05	<0.25
2	3.77	3.25	27	<0.05	<0.25
3	1.77	1.59	28	<0.05	<0.25
4	<0.05	<0.25	29	4.44	4.1
5	6.55	7.26	30	1.78	1.89
6	15.48	15.06	31	0.2	<0.25
7	7.66	7.19	32	0.29	<0.25
8	5.93	5.97	33	0.61	0.5
9	0.65	0.57	34	1.3	1.37
10	0.99	0.68	35	0.69	0.64
11	1.72	1.8	36	<0.05	<0.25
12	3.45	3.36	37	1.92	2.06
13	2.16	2.15	38	2.62	2.57
14	2.97	2.45	39	0.99	0.96
15	0.73	0.55	40	2.01	1.79
16	0.79	0.81	41	3.32	2.96
17	1.14	1.44	42	1.75	1.53
18	0.33	<0.25	43	0.46	0.38
19	0.61	0.58	44	1.37	1.09
20	0.43	0.25	45	1.34	1.16
21	0.31	<0.25	46	1.19	1.05
22	3.06	3.02	47	1.3	1.14
23	0.52	0.44	48	1.07	0.69
24	1.45	1.38	49	1.1	0.81
25	<0.05	<0.25	50	1	0.77

^a^ Kit LFD: RIDA®QUICK DON RQS ECO in combination with the RIDA^®^SMART APP.

## Data Availability

The original contributions presented in this study are included in the article. Further inquiries can be directed to the corresponding author(s).
